# Development of the Diabetes Injection Device Experience Questionnaire (DID-EQ) and Diabetes Injection Device Preference Questionnaire (DID-PQ)

**DOI:** 10.1186/s41687-018-0068-z

**Published:** 2018-09-12

**Authors:** Louis S. Matza, Kristina S. Boye, Katie D. Stewart, Rosirene Paczkowski, Jessica Jordan, Lindsey T. Murray

**Affiliations:** 1 Patient-Centered Research, Evidera, 7101 Wisconsin Avenue, Suite 1400, Bethesda, MD 20814 USA; 2 Eli Lilly and Company, Indianapolis, IN USA

**Keywords:** Type 2 diabetes, Injection device, GLP-1 receptor agonist, Patient-reported outcomes measures, PRO, Concept elicitation, Qualitative research, Treatment satisfaction, Treatment preference

## Abstract

**Background:**

Previous research has examined patient perceptions of insulin injection devices. However, injectable medications other than insulin are increasingly used to treat type 2 diabetes, including GLP-1 receptor agonists. No patient-reported outcome (PRO) instruments have been developed taking into account the experiences of patients using newer injection devices, which are often different from devices used for insulin. Therefore, the purpose of this qualitative study was to develop two draft PRO instruments focusing on patients’ experiences with these newer injection devices (one instrument assessing perceptions of a single injection device, and another assessing preferences between two devices).

**Methods:**

Questionnaire development proceeded in six steps: literature review, interviews with six device experts, concept elicitation interviews with patients (*N* = 32), preliminary translatability assessment, cognitive interviews with patients (*N* = 20), and final translatability assessment.

**Results:**

Literature review and expert interviews were conducted to inform a concept elicitation interview guide. In concept elicitation in the US, UK, and Germany, patients with type 2 diabetes reported a range of injection features that influenced their perceptions of non-insulin injection devices (e.g., requirements for preparation of the medication/device, issues related to the needle, ease-of-use, portability). Two draft “item pools” were developed based on the literature review, expert interviews, and concept elicitation results. In cognitive interviews, patients recommended minor revisions and indicated that the draft instruments were generally clear, comprehensible, and relevant to their experience with non-insulin injectable medication. The instruments were refined based on the cognitive interviews and translatability assessment, resulting in two questionnaires.

**Conclusions:**

The various steps of qualitative research support the content validity of these new PRO instruments, which are the first developed specifically to assess perceptions of non-insulin injection delivery systems. Despite some overlap with insulin-focused questionnaires, the new instruments are distinct from previous instruments (omitting content that would not be relevant to patients receiving non-insulin injectable treatment, while including content that is not included in the insulin focused instruments). This qualitative research yielded two draft questionnaires that are grounded in patient perceptions and ready for psychometric validation studies with larger samples of patients with type 2 diabetes.

## Background

A range of patient-reported outcomes instruments (PROs) are available for assessing patient perceptions of treatment for type 2 diabetes. When added to clinical outcomes such as HbA1c (average blood glucose level over the past 2 to 3 months), PROs can provide a more thorough picture of patients’ experience with type 2 diabetes and its treatments. In addition, it is important to understand the patient perspective because greater treatment satisfaction can lead to better treatment adherence [1–4], which has a positive impact on health outcomes [5, 6]. Several PRO measures have been developed specifically to assess perceptions of insulin treatment, including the injection process [7–10]. However, injectable medications other than insulin are increasingly used to treat type 2 diabetes [11–13]. PRO instruments designed to assess perceptions of insulin treatment are not necessarily appropriate for the newer treatments, which may differ from insulin in multiple aspects of treatment administration and the injection device.

Therefore, the purpose of this study was to conduct qualitative research to support the development of two new questionnaires focusing on patients’ perceptions of injection devices used with non-insulin treatments for type 2 diabetes. The available non-insulin injectable medications for type 2 diabetes include pramlintide [14] and several medications in the class of glucagon-like peptide-1 receptor agonists (GLP-1 RAs) [11]. The GLP-1 RAs are often recommended as part of combination therapy when oral medication alone does not result in sufficient control of blood glucose levels [15–17]. GLP-1 RAs can be effective in lowering blood glucose levels and body weight, with a low risk of hypoglycemia [11, 18–20]. The most commonly reported adverse events are gastrointestinal side effects [19–21].

Despite these similarities in efficacy and safety, GLP-1 RAs vary in their injection devices and related treatment administration attributes. For example, the injection devices differ in size, requirements for needle handling, and multiple versus single use [22–28]. In addition, some GLP-1 RAs require the patient to reconstitute and/or mix the medication in the injection device prior to injection [23, 25] while others do not have these requirements [22, 24, 26–28]. Furthermore, the frequency varies widely as some GLP-1 RAs are injected every day [22, 26, 27] while others are injected once weekly [23–25, 28]. These differences in the injection device and injection process could impact patients’ quality of life and preference among treatments. Although these differences among treatments could be important to patients, there are no previously available questionnaires designed to assess patient experience or preference among these new injection devices.

Previous PRO instruments designed to assess treatment experiences among patients with type 2 diabetes were not developed while considering differences among these newer injectable treatments. The majority of the existing diabetes-specific measures are focused on insulin treatment and include concepts that are not applicable to treatment with most of the GLP-1 RAs, such as dose selection and adjustment, measuring blood glucose or glycemic control, and planning food or physical activities around treatment. Examples of these instruments include the Insulin Delivery System Rating Questionnaire (IDSRQ) [9], the Insulin Delivery System Questionnaire (IDSQ) [29, 30], Insulin Injection Preference Questionnaire (IIP-Q) [10], the Insulin Pens Questionnaire – Evaluation (IPQ-E) [31]; Insulin Treatment Satisfaction Questionnaire (ITSQ) [7], Patient Satisfaction with Insulin Therapy (PSIT) [32, 33], Treatment-Related Impact Measures for Diabetes and Diabetes Devices (TRIM-D and TRIM-D Device) [8, 34]. Previously existing measures also omit concepts that could be important when assessing patient experiences with injection devices such as preparing the device/medication for use (missing from IPQ-E, ITSQ, and PSIT), confidence in providing the correct dose (missing from ITSQ, PSIT), the size of the needle (missing from IDSQ, IPQ-E, TRIM-D/TRIM-D Device), or the time to prepare and inject the medication (missing from IDSQ, IIP-Q, IPQ-E, ITSQ, TRIM-D/TRIM-D Device). More general instruments, such as the Diabetes Treatment Satisfaction Questionnaire (DTSQ) [35], are useful across a wide range of treatments, but may not include specific injection device characteristics. Importantly, none of these measures were developed based on perceptions of patients treated with GLP-1 RAs, and therefore may not reflect the key features of these new treatments that are most important to patients treated with these newer injectable medications.

In contrast to these previously developed instruments, the content of the two PRO measures developed in the current study is derived directly from the input of patients who have experience with these newer treatments, including a range of the available GLP-1 RAs. This qualitative study included two phases of interviews with patients who had been treated with non-insulin injectable medication for type 2 diabetes. The aim of this qualitative research was to gain an understanding of patients’ experiences with these newer injection delivery systems, which would ensure that concepts important to these patients would be included in two new questionnaires. The first questionnaire was designed to assess perceptions of a single injection device, while the second asks patients to report preferences between two devices.

## Methods

### Overview

**Fig. 1:**
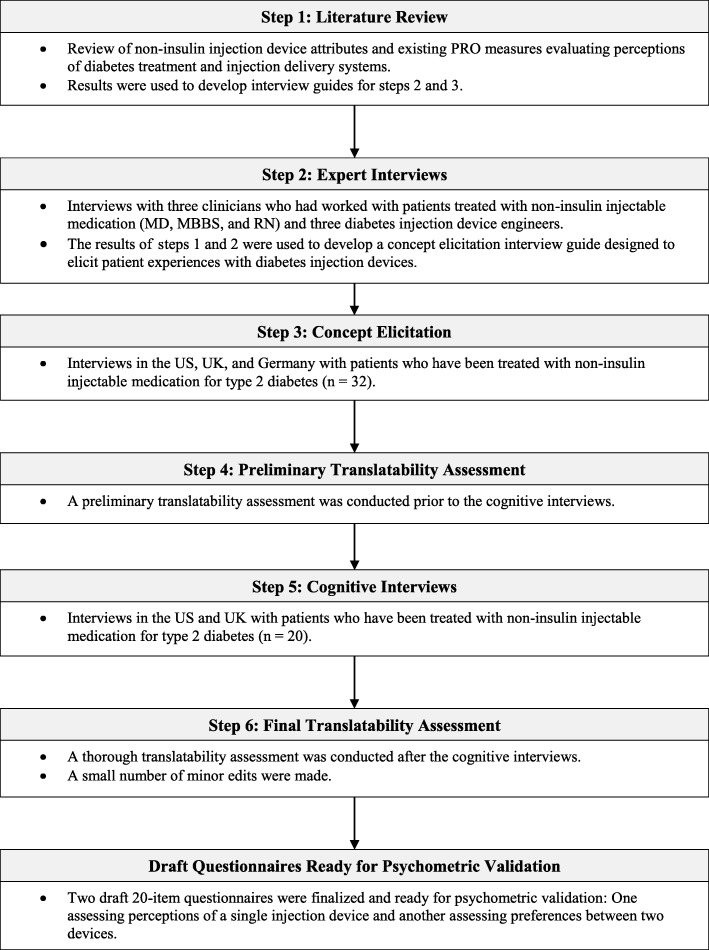
Summary of Instrument Development for the Diabetes Injection Device Experience Questionnaire and Preference Questionnaire

Development of these draft questionnaires proceeded in a series of six steps summarized in Fig. 1. The first step was literature review, designed to inform development of interview guides for use in subsequent discussions with clinicians, device experts, and patients. This literature review focused on identifying attributes of non-insulin injection devices that could be important to patients, as well as previously developed PRO instruments designed to assess content that could be relevant to diabetes injection devices. Sources of information included the instructions for use of every available non-insulin injection device, as well as published articles on these medications and diabetes-specific PRO measures identified via PubMed.

In step 2, six experts were interviewed about diabetes injection devices by telephone or in person. These experts included three clinicians who had worked with patients treated with non-insulin injectable medication (MD, MBBS, and RN) and three diabetes injection device engineers. These interviews focused on factors differentiating among diabetes injections devices; strengths and inconveniences of non-insulin injection devices that were available at the time; ways that injection devices may influence treatment decisions; device attributes clinicians consider when selecting a device; and execution for development of non-insulin injection devices in the future. Key factors said to differentiate among devices included device preparation requirements, reconstitution, adjustable dose selection (compared to fixed dose devices), patient confidence in using the device, needle handling requirements, needle visibility, needle safety, capping, disposal, overall ease of use, and single-use versus multiple-use devices. The results of steps 1 and 2 were used to develop a concept elicitation interview guide designed to elicit patient experiences with diabetes injection devices.

In step 3, concept elicitation interviews were conducted with patients in the US, UK, and Germany. Interviews focused on experiences with devices used to administer non-insulin injectable medication. Qualitative information gathered during these interviews was used to generate the initial item pools: one set of items for evaluating patient perceptions of a single injection device (“single device questionnaire”) and a parallel set of items for assessing preference between two injection devices (“preference questionnaire”). In step 4, the draft item pools were evaluated in a preliminary translatability assessment (performed by a member of the Leadership Team of the International Society for Pharmacoeconomics and Outcomes Research [ISPOR] Patient-Reported Outcomes [PRO] Task Force for Translation and Linguistic Validation), and edits were made to the word choice and item structure.

In step 5, the draft item pools were evaluated in cognitive interviews with type 2 diabetes patients in the US and UK. Each participant was asked to complete the single device questionnaire. Participants who had experience with more than one injection device were also asked to complete the preference questionnaire, while participants who had used only one injection device were asked to review (but not complete) the preference questionnaire. Respondents were queried on how they interpreted, understood, and responded to the questionnaires, with specific focus on interpretation of instructions, items, and response options. In addition to reviewing the content of the items, participants were asked if there were relevant attributes of their injection devices that were not addressed in the instrument. The item pools were edited based on patient feedback during the cognitive interviews and a subsequent final translatability assessment (step 6; performed by a member of the Leadership Team of the ISPOR PRO Task Force for Translation and Linguistic Validation), yielding two draft questionnaires ready for item reduction and psychometric validation in a subsequent study with a larger sample to be reported elsewhere.

### Qualitative research participants

Patients with type 2 diabetes were interviewed for the concept elicitation and cognitive interview steps of instrument development. All participants in both of these steps were required to be (1) diagnosed with type 2 diabetes by a recognized medical professional; (2) at least 18 years of age at the time of the interview; (3) able to provide proof of type 2 diabetes diagnosis; (4) residing in the US or UK (or Germany for concept elicitation interviews); (5) able to read, speak, and understand the native language of their interview location; (6) able and willing to give written informed consent prior to study entry; and (7) able to complete the protocol requirements.

Participants in the concept elicitation interviews were required to be either currently treated for type 2 diabetes with a non-insulin injectable medication (i.e., one of the GLP-1 receptor agonists or pramlintide) or have discontinued treatment with one of these non-insulin injectable medications within the last 6 months. Participants in the cognitive interviews were required to be currently receiving treatment with a non-insulin injectable medication. Patients who had discontinued a non-insulin injectable medication were eligible for concept elicitation interviews because their perceptions of a previously used device could have provided useful insight, particularly if they discontinued the medication because of the device. However, for the cognitive interviews, it was important that patients were currently using a relevant device so that they could complete questionnaire items based on current perceptions, as indicated in the questionnaire instructions. Participants were eligible for either phase regardless of whether they received insulin in addition to the non-insulin medication.

Potential participants were excluded if the participant (1) had a cognitive impairment, hearing difficulty, visual impairment, acute psychopathology, or insufficient knowledge of the interview language that in the opinion of the investigator/interviewer could interfere with his or her ability to provide written consent and complete an interview; (2) was unable to recall any characteristics about their injection device; (3) was currently diagnosed with gestational diabetes instead of type 2 diabetes; and/or (4) receiving a GLP-1 receptor agonist for weight loss rather than type 2 diabetes.

All participants were recruited via newspaper and online advertisements. Concept elicitation participants were recruited in 15 cities across the United States (Washington, DC; Omaha, NE; Tampa, FL; Phoenix, AZ; San Diego, CA; San Antonio, TX; Dallas, TX; Houston, TX; Birmingham, AL; Atlanta, GA; and Indianapolis, IN), the UK (Edinburgh and London), and Germany (Hamburg and Berlin). Cognitive interview participants were recruited in 11 cities across the United States (Washington, DC; Baltimore, MD; Omaha, NE; San Diego, CA; San Antonio, TX; Dallas, TX; Houston, TX; Birmingham, AL; Atlanta, GA; Indianapolis, IN) and the UK (London). All potential participants who responded to the advertisements were screened by telephone using a standardized screening script.

### Data collection: Two qualitative methods

This research followed standard methods for qualitative research used to support PRO instrument development, including both concept elicitation and cognitive interviews [36–39]. Concept elicitation interviews in step 3 were conducted with patients with type 2 diabetes to inform the content of the instrument. These interviews were conducted using a semi-structured interview guide developed based on the results of the literature review (step 1) and interviews with clinicians and injection device engineers (step 2). The interview focused on patients’ perceptions and experiences of non-insulin injection devices associated with treatment for type 2 diabetes. Each interview began with open-ended questions to elicit spontaneous comments unprompted by suggestions of any specific device attributes (e.g., Tell me about your experience with this device. What do/did you like/dislike about the device? Are there features of the device that make it convenient/inconvenient/easy/difficult to use?). After patients answered these open-ended questions, a series of targeted questions were asked about specific injection device attributes the patients had not yet mentioned (e.g., steps for preparing the medication/device prior to injection, frequency of injections, portability, comfort/discomfort, disposal, storage, issues associated with the needle, dose selection, time required for the injection process).

Cognitive interviews in step 5 evaluated the draft questionnaires in terms of ease of use, clarity, comprehensibility, comprehensiveness, redundancy, and relevance. Respondents first completed the draft questionnaires, and were then interviewed according to a structured interview guide that focused on evaluating patients’ understanding of the instructions, questions, response options, and recall period. The participants were asked to describe how they understood the instrument’s instructions, how they interpreted each item, and how they selected a response.

All methods and materials were approved by an Independent Review Board [Ethical and Independent Review Services (E&I), E&I study numbers 14173–01 and 15099–01], and all patients provided written informed consent prior to completing any study procedures. Interviews were audio recorded and transcribed so that the qualitative data could be coded and analyzed (and in the case of the German interviews, translated prior to coding).

### Qualitative data analysis

Qualitative data from the concept elicitation and cognitive interviews were analyzed using ATLAS.ti software and a content analysis approach [40]. A coding dictionary of themes, concepts, and terms was developed for use in the qualitative analysis. For concept elicitation interviews, the coding dictionary included concepts relating to participants’ experience with their injection devices and injection device attributes. For cognitive interviews, the dictionary included codes related to item clarity, item comprehension, ease of completing the items, comprehensiveness of the instrument, and appropriateness of the response scales.

For both concept elicitation and cognitive interviews, two staff members independently coded the first interview transcript from each interview step. A post-coding comparison and reconciliation occurred, and all codes were compared, discussed, and reconciled wherever differences occurred. After agreement between the two coders was sufficient based on determination by a senior staff member, one coder coded the remaining transcripts with a quality review by senior staff members.

Participant quotes were categorized by thematic code, and saturation was documented by tracking concepts in saturation grids. Saturation is defined as the point at which no substantially new themes, concepts, or terms are introduced as additional interviews are conducted [37].

## Results

### Steps 1 and 2: Literature review and expert interviews

A total of eight injection devices were identified for non-insulin medications used to treat diabetes: exenatide extended release single-dose tray, exenatide once weekly pen, exenatide twice daily pen, dulaglutide pen, liraglutide pen, albiglutide pen, pramlintide pen, and lixisenatide pen. Based on published literature, drug labels, and device instructions for use, key attributes that differ among these eight non-insulin injection devices included frequency of injections, visibility of the needle, single use/reusable, requirements for reconstitution (i.e., mixing the medication), waiting time during reconstitution, wait time after removing from refrigerator, requirements for timing of administration (including with or without meals), and requirements for attaching/handling the needle [11, 17–21, 41].

The literature review also identified a range of PRO measures that were previously developed to assess perceptions of diabetes treatment. Some of these instruments include items assessing perceptions of the insulin injection process, and these items were considered relevant for development of the new questionnaires. Previous relevant PRO measures include the Diabetes Treatment Satisfaction Questionnaire (DTSQ) [35], Insulin Delivery System Questionnaire (IDSQ) [29], Insulin Injection Preference Questionnaire (IIP-Q) [10], Insulin Treatment Satisfaction Questionnaire (ITSQ) [42], and the Treatment Related Impact Measures for Diabetes and Diabetes Devices (TRIM-D) [34], among others.

Three clinicians and three injection device engineers also provided input regarding differences among available injections devices for non-insulin medications, including strengths and inconveniences of each injection process, device attributes that clinicians consider when choosing which medication to prescribe, and expectations for future non-insulin injection devices. Key factors suggested to differentiate among injection devices included device preparation requirements, reconstitution procedures, adjustable dose selection (compared to fixed dose devices), patient confidence in preparing and using the device, needle handling requirements, needle visibility, needle safety, capping of the devices, and disposal of the devices. The results of the literature review and expert interviews were used to inform the development of the concept elicitation interview guide for step 3.

### Steps 3 and 4: Concept elicitation interviews and preliminary translatability assessment

A total of 32 patients completed concept elicitation interviews in the UK (*n* = 14), the US (*n* = 11), and Germany (*n* = 7) (see demographic and clinical characteristics in Table 1). Thirty participants discussed devices associated with GLP-1 receptor agonists that they were currently using. In addition to discussing current devices, two of these 30 participants also discussed a GLP-1 receptor agonist device that they had discontinued in the past 6 months. An additional two participants were not currently treated with a non-insulin injectable medication. Instead, these two participants discussed GLP-1 receptor agonists that they had discontinued within the past 6 months. The most commonly used GLP-1 receptor agonists in this sample were liraglutide (*n* = 11), exenatide (*n* = 5), exenatide extended release (*n* = 4), and exenatide extended release single-dose tray (*n* = 4).

**Table 1:** Participant Characteristics (Concept Elicitation)

Characteristic	US	UK	Germany	Total Sample^a^
*N*	11	14	7	32
Mean Age (years) (SD)	56.91 (11.4)	59.57 (14.0)	67.43 (5.4)	60.38 (12.0)
Gender				
Female	7 (63.6%)	6 (42.9%)	3 (42.9%)	16 (50.0%)
Male	4 (36.4%)	8 (57.1%)	4 (57.1)	16 (50.0%)
Ethnic/Racial Background (*n*, %)				
White	4 (36.4%)	12 (85.7%)	7 (100.0%)	23 (71.9%)
Mixed	1 (9.1%)	0 (0.0%)	0 (0.0%)	1 (3.1%)
Asian	1 (9.1%)	0 (0.0%)	0 (0.0%)	1 (3.1%)
Black	4 (36.4%)	2 (14.3%)	0 (0.0%)	6 (18.8%)
Other^b^	1 (9.1%)	0 (0.0%)	0 (0.0%)	1 (3.1%)
Marital Status (*n*, %)				
Single	1 (9.1%)	4 (28.6%)	0 (0.0%)	5 (15.6%)
Married/Living with partner	6 (54.5%)	7 (50.0%)	4 (57.1%)	17 (53.1%)
Other^c^	4 (36.4%)	3 (21.4%)	3 (42.9%)	10 (31.3%)
Employment Status (*n*, %)^d^				
Full-time work	5 (45.5%)	2 (14.3%)	1 (14.3%)	8 (25.0%)
Part-time work	1 (9.1%)	2 (14.3%)	0 (0.0%)	3 (9.4%)
Other^e^	5 (45.5%)	10 (71.4%)	6 (85.7%)	21 (65.6%)
Education Level (*n*, %)				
University degree	5 (45.5%)	3 (21.4%)	2 (28.6%)	10 (31.3%)
No University degree	6 (54.5%)	11 (78.6%)	5 (71.4%)	22 (68.7%)
Interview Type				
In-Person (*n*)	1 (9.1%)	14 (100.0%)	1 (14.3%)	16 (50.0%)
Telephone (*n*)	10 (90.9%)	0 (0.0%)	6 (85.7%)	16 (50.0%)
Medication History				
Current GLP-1	7 (63.6%)	11 (78.6%)	7 (100.0%)	25 (78.1%)
Discontinued GLP-1	0 (0.0%)	2 (14.3%)	0 (0.0%)	2 (6.3%)
Current GLP-1 and Insulin	4 (36.4%)	1 (7.1%)	0 (0.0%)	5 (15.6%)
Current GLP-1 RAs				
Liraglutide	3 (27.3%)	3 (21.4%)	4 (57.1%)	10 (31.3%)
Exenatide twice daily	0 (0.0%)	4 (28.6%)	1 (14.3%)	5 (15.6%)
Exenatide extended release tray	1 (9.1%)	1 (7.1%)	2 (28.6%)	4 (12.5%)
Lixisenatide	0 (0.0%)	4 (28.6%)	0 (0.0%)	4 (12.5%)
Exenatide extended release pen	3 (27.3%)	0 (0.0%)	0 (0.0%)	3 (9.4%)
Dulaglutide	2 (18.2%)	0 (0.0%)	0 (0.0%)	2 (6.3%)
Albiglutide	2 (18.2%)	0 (0.0%)	0 (0.0%)	2 (6.3%)
Discontinued GLP-1 RAs^f^				
Liraglutide	1 (9.1%)	1 (7.1%)	0 (0.0%)	2 (6.3%)
Exenatide extended release pen	1 (9.1%)	1 (7.1%)	0 (0.0%)	2 (6.3%)

^a^This table includes data for the 32 participants who provided proof of medication, were eligible, and completed a satisfactory interview according to the interview guide. Two additional participants were enrolled in the study but excluded from the analysis sample because of deviations from the interview guide

^b^Other ethnic/racial background includes: Hispanic (total: *n* = 1; US: *n* = 1)

^c^Other marital status includes: Divorced (total: *n* = 7; US: *n* = 4; Germany: *n* = 3); Separated (total: *n* = 2; UK: *n* = 2), Widowed (total: *n* = 1; UK: *n* = 1)

^d^Percentages for employment status in the US sum to 100.1% due to rounding

^e^Other employment status includes: US: Retired (*n* = 3), Retired and disabled (*n* = 2); UK: Retired (*n* = 9), Homemaker (*n* = 1); Germany: Retired (*n* = 5), Retired and disabled (*n* = 1)

^f^GLP-1 RAs discontinued within the past 6 months. No patients reported discontinuing any GLP-1 RAs other than liraglutide and exenatide once weekly

During the interviews, the participants reported a wide range of attributes of injection devices and injection processes that they liked and disliked. Positive and negative injection attributes are listed in Tables 2 and 3, respectively, along with frequencies of participants reporting each attribute and examples of quotes for each attribute. The attributes included injection preparation, injection comfort, the needle, dose delivery confidence/confirmation, injection frequency, selecting a dose, disposal, time requirements, size, injection site, design/appearance, storage, portability, discreetness, and confidence in appropriate device use. Many of the attributes were viewed positively by some respondents and negatively by others. No new important concepts or themes emerged in the final five interviews. Therefore, it was determined that concept saturation was reached, and no additional concept elicitation interviews were needed. The concepts and terms identified in these interviews were used to develop item pools for the single device questionnaire and the preference questionnaire, thus ensuring that the content of the questionnaires was grounded in patients’ descriptions of their experience with non-insulin injectable medication and the associated injection devices. Instructions and response options were also drafted so that they could be tested in the subsequent cognitive interview study.

**Table 2:** Positive Perceptions of Injection Device Attributes (*N* = 32)

Injection Device Attribute	Number of Patients Reporting Positive Perceptions			Example Quotations
	Spontaneous^a^	Prompted^b^	Total^c^	
Injection preparation	21	9	30	You just put the needle on top, screw the needle on, and you set the—the dial, and you press the button. So, it’s very easy.
Injection comfort	10	18	28	… it was painless.
Needle	13	15	28	I like that I’m not having to put the needle in myself.
Dose delivery confidence/confirmation	12	16	28	It’s premeasured and all I have to do is make sure that it’s mixed and inject.
Injection frequency	8	17	25	I do not have to inject the [medication] every day, but only once a week. I think that is just great.
Selecting dose	11	14	25	The pen does it itself…I like that. I wouldn’t like to select how much I needed each day as I believe you have to with some insulins.
Disposal	3	21	24	Everything is fine on the way you take it apart and dispose of it.
Time requirements	5	19	24	It’s very quick and easy.
Size	7	16	23	It’s compact in size, you can carry it in your pocket.
Design/appearance	3	19	22	There’s bright colors on there as well, so obviously it’s easy to notice or easy to spot. I know it’s separate from my other medication.
Storage	6	16	22	It’s, you know, it’s a little compact, so it doesn’t take much space.
Portability	6	15	21	It is easy to carry with me.
Dosing schedule	11	9	20	It is routine now and when it is routine all that becomes automatic and so I actually do not forget because it is always the next item on the agenda after breakfast.
Injection timing/flexibility	2	17	19	It gives you a lot more control, a lot more freedom of not being able to take it if you’re not—you know, when it’s convenient for you.
Discreetness	0	11	11	I don’t want everyone to know what I’m taking. But then it’s very discreet that it looks like I might be using it just to write something, but it’s not.
Confidence in appropriate device usage	7	2	9	I liked how the little, uh, thing popped out of the end of the pen, the little pump thing, pops out at the end of the pen, I really like that because it showed me that I did it correctly.

^a^Concepts introduced by participants without prior mention by the interviewer

^b^Concepts endorsed by participants in response to prompting from the interviewer

^c^The total number of participants endorsing a concept either spontaneously or in response to prompting

**Table 3:** Negative Perceptions of Injection Device Attributes (*N* = 32)

Injection Device Feature	Number of Patients Reporting Negative Perceptions			Example Quotations
	Spontaneous^a^	Prompted^b^	Total^c^	
Needle	14	4	18	I think I don’t like the fact that after you mix it up, you now have to screw the needle on top of it.
Portability	10	6	16	Well, like I said, I don’t carry it with me, I—I do it at my house because it is too big to carry.
Discreetness	4	11	15	This was a little bit bigger where it was much more difficult to take it without someone seeing it or, you know, realizing what I’m doing.
Storage	9	6	15	Storage, um, the box takes up a little bit of room in my refrigerator, I would rather it be a smaller box...
Injection preparation	12	2	14	When you’re mixing them you have to be careful, you know, how you mix it…I wish I didn’t have to go through the stages of, you know, mixing it and, um, shaking the device, and making sure it’s well diluted, you know. It’s a waste of time.
Confidence in appropriate device usage	10	2	12	Any little mistake could like, you know, it could result to like, not proper administration of dosage.
Size	9	2	11	It’s a little bit cumbersome, a little bit too big.
Injection comfort	9	2	11	It’s getting plenty sore, I’ve got to try to find a different area every week.
Multiple parts	9	1	10	I’d prefer if it was just together.
Dose delivery confidence/confirmation	6	3	9	I will say I wonder sometimes if it all goes in because they’ll be like – when I pull it out sometimes there will be liquid at the end of it. So yeah, I guess sometimes I wonder if it’s all going in.
Dosing schedule	3	4	7	It’s a bad thing because it’s not something you can do in a hurry or if you’re on the run.
Injection timing/flexibility	2	5	7	There is a strict time window, you must take it in the hour before you eat a large meal. That can be—that can be problematic.
Injection site	4	3	7	You can only do it in certain areas of your body…The part that bothers me is that it can get very sensitive so you have to find a spot that’s not sensitive.

^a^Concepts introduced by participants without prior mention by the interviewer

^b^Concepts endorsed by participants in response to prompting from the interviewer

^c^The total number of participants endorsing a concept either spontaneously or in response to prompting

The draft item pools and instructions were reviewed in a preliminary translatability assessment (step 4), leading to minor wording revisions. The two resulting item pools (i.e., the single device questionnaire and the preference questionnaire) ready for the cognitive interviews each contained 16 items on specific attributes (injection preparation, comfort/pain, confidence in dose delivery, confidence in correct/appropriate use, needle size, needle use, injection frequency, injection timing/flexibility, time required for the injection process, size, portability, storage, selecting dose, disposal) and three global items assessing satisfaction, ease of use, and convenience. Depending on the phrasing, each item of the single device questionnaire was answered using a 4-point response option scale assessing satisfaction, ease of use, confidence, or agree/disagree. Each item of the preference questionnaire had a 5-point response scale on which respondents indicate which of two devices is preferred.

### Steps 5 and 6: Cognitive interviews and final translatability assessment

A total of 20 patients with type 2 diabetes participated in cognitive interviews in the US (*n* = 15) and UK (*n* = 5) (see demographic and clinical characteristics in Table 4). Each participant was asked to complete the single device questionnaire. Participants who had experience with more than one non-insulin injection device were also asked to complete the preference questionnaire in order to compare two devices. Participants who had experience with only one non-insulin injection device were asked to review, but not complete, the preference questionnaire.

**Table 4:** Participant Characteristics (Cognitive Interviews)

Characteristic	US	UK	Total Sample
*N*	15	5	20
Mean Age (years)	64.7 (7.5)	57.0 (7.8)	62.8 (8.1)
Gender (*n*, %)			
Female	10 (66.7%)	2 (40.0%)	12 (60.0%)
Male	5 (33.3%)	3 (60.0%)	8 (40.0%)
Ethnic/Racial Background (*n*, %)			
White	11 (73.3%)	4 (80.0%)	15 (75.0%)
Mixed	0 (0.0%)	0 (0.0%)	0 (0.0%)
Asian	0 (0.0%)	1 (20.0%)	1 (5.0%)
Black	1 (6.7%)	0 (0.0%)	1 (5.0%)
Other^a^	3 (20.0%)	0 (0.0%)	3 (15.0%)
Marital Status (*n*, %)			
Single	1 (6.7%)	2 (40.0%)	3 (15.0%)
Married/Living with partner	12 (80.0%)	2 (40.0%)	14 (70.0%)
Other^b^	2 (13.3)	1 (20.0%)	3 (15.0%)
Employment Status (*n*, %)			
Full-time work	2 (13.3%)	2 (40.0%)	4 (20.0%)
Part-time work	4 (26.7%)	1 (20.0%)	5 (25.0%)
Other^c^	9 (60.0%)	2 (40.0%)	11 (55.0%)
Education Level (*n*, %)			
University degree	7 (46.7%)	2 (40.0%)	9 (45.0%)
No University degree	8 (53.3%)	3 (60.0%)	11 (55.0%)
Interview Conducted			
In-Person (*n*)	0 (0.0%)	3 (60.0%)	3 (15.0%)
Telephone (*n*)	15 (100.0%)	2 (40.0%)	17 (85.0%)
Current Medication			
GLP-1	15 (100.0%)	5 (100.0%)	20 (100.0%)
Insulin	2 (13.3%)	0 (0.0%)	2 (10.0%)
Pramlintide	1 (6.7%)	0 (0.0%)	1 (5.0%)
Current GLP-1			
Exenatide extended release (either pen or tray)	7 (46.7)	0 (0.0%)	7 (35.0%)
Liraglutide	4 (26.7)	2 (40.0%)	6 (30.0%)
Dulaglutide	3 (20.0%)	0 (0.0%)	3 (15.0%)
Lixisenatide	0 (0.0%)	2 (40.0%)	2 (10.0%)
Exenatide twice daily	0 (0.0%)	1 (20.0%)	1 (5.0%)
Albiglutide	1 (6.7%)	0 (0.0%)	1 (5.0%)
Pramlintide	0 (0.0%)	0 (0.0%)	0 (0.0%)

^a^Other ethnic/racial background includes: Hispanic (total: *n* = 3; US: *n* = 3)

^b^Other marital status includes: Separated (total: *n* = 3; UK: *n* = 1; US: *n* = 2)

^c^Other employment status includes: Retired (total: *n* = 10; US: *n* = 8; UK: *n* = 2); homemaker (total: *n* = 1; US: *n* = 1)

Most participants reported that the questionnaires were clear and easy to complete. Some minor revisions were made to specific items based on participant feedback. For example, participants had difficulty with the initial wording of the item on injection pain (originally phrased as “the injections are painful”) and offered suggestions for revisions. This item was re-worded twice during the cognitive interview phase. All participants completing the final version of the item (“How often do you experience pain when injecting medication with this device?”) demonstrated that they understood the item as intended without difficulty. The item on difficulty with dose selection (“How difficult is it to select the correct dose for each injection?”) was not applicable to all participants because many non-insulin injection devices do not require patients to select a dose. The phrasing of this item was revised to ask about frequency to allow participants to answer the item even if their device does not require dose selection (“How often do you have difficulty selecting the correct dose with your injection device?”). Feedback from participants suggested that the item on disposal of the device and its parts was confusing because it assessed two constructs. Therefore this item was split into separate questions asking about disposal of the injection device (“How difficult is it to dispose of the injection device?”) and disposal of the device parts (“How difficult is it to dispose of materials associated with the injection device, such as the cap and needle?”). Two items were added based on recommendations from participants. These additional items assessed anxiety and difficulty keeping the injection device and medication at the correct temperature when it is necessary to inject away from home.

The draft questionnaires were updated several times during the cognitive interview study based on this feedback from participants. In total, there were four rounds of interviews, each with slightly updated versions of the questionnaires. In the final round of interviews with three participants completing the final versions of the questionnaires, participants understood the questionnaire items as intended and were able to answer the items without difficulty.

A thorough translatability assessment was conducted after the cognitive interviews were completed. A small number of comments suggested minor wording changes to the questionnaire. None of these changes altered the content or meaning of the items. For example, the item “how often do you have anxiety about using your injection device?” was revised to “how often do you feel anxious about using your injection device?”, as this phrasing may be more culturally appropriate for some translations.

After edits based on the translatability assessment and cognitive interviews, the resulting versions of the single device questionnaire and preference questionnaire each contained 20 items assessing the following concepts: preparation for use, using the needle, disposal of injection device, disposal of materials associated with the injection device, fitting the injection into routine, storage, portability, storage temperature, confidence in dose delivery, confidence in device use, needle size, device size, injection frequency, time required for preparation and injection, selecting dose, pain, anxiety, and three global items on satisfaction, ease of use, and convenience.

In the instructions for a PRO instrument, the recall period is an important part of the instructions [43, 44]. Because these new questionnaires focus on perceptions of injectable treatment rather than symptoms or disease impact that tend to fluctuate over time, the items focus on current perceptions rather than an extended recall period. The instructions of the single device questionnaire begin with “Please select one response for each item to indicate how you currently feel...”. For the preference questionnaire, the word “currently” could have been confusing because respondents need to consider both a current and previous medication. Therefore, the instructions of the preference questionnaire do not use the word “currently,” but instead implicitly ask for current preference (“Please select one response for each item to indicate which of the two injection devices you prefer.”).

## Discussion

Taken together, the various steps of qualitative research support the content validity of these two new PRO instruments to assess perceptions of non-insulin injection devices and the injection process. In the multi-country concept elicitation study, patients who have been treated with non-insulin injectable medication for type 2 diabetes reported a wide range of injection device features that influenced their perceptions of the injection experience. The patient perspective elicited in these interviews, in combination with clinician input and published literature, was used to generate content of the instruments. Subsequent cognitive interviews with additional patients indicated that the draft instruments were clear, comprehensible, and relevant to patients who had been treated with non-insulin injectable medications. The questionnaires were then refined based on these cognitive interviews, as well as a translatability assessment. These steps of instrument development resulted in two draft questionnaires that are grounded in patient perceptions and ready for psychometric validation studies with larger samples of patients with type 2 diabetes.

In addition to supporting instrument development, results of the concept elicitation interviews highlight aspects of injectable treatment that are important to patients (listed in Tables 2 and 3). Nearly all injection device features were viewed favorably by some respondents and unfavorably by others, usually depending on whether the device features were perceived as contributing to ease-of-use. For example, some patients viewed the relatively simple injection preparation as a positive feature of their injection device (e.g., 301–025: “I get my device, put the needle in, click it to the dosage amount, and then inject. It’s very easy.”). However, other medications require more steps prior to injecting the medication, and patients receiving these treatments viewed injection preparation as a negative feature (e.g., 101–003: “I just wish it was already mixed up… I wish I didn’t have to go through the stages of mixing it and shaking the device, and making sure it’s well diluted. It’s a waste of time.”). While injection device features are likely to be a lower priority than efficacy and safety, these factors could contribute to patient satisfaction and adherence, which could have an impact on treatment outcomes [6]. Therefore, in clinical settings, it may be important to consider these device and injection process features when considering treatments for individual patients.

Although these are the first PRO instruments developed based on perceptions of patients treated with non-insulin injectable medications for type 2 diabetes, there is overlap between these new instruments and instruments developed in samples of insulin-treated patients. Several constructs in the new instruments are also assessed by insulin-focused PRO measures (e.g., general ease-of-use, size of device, physical discomfort, confidence that the device delivers the correct dose) [7, 9, 10, 29, 33, 34]. Despite this overlap, the new instruments diverge from these previous insulin-focused instruments in important ways. A useful example is the 8-item TRIM-D Device instrument, which focuses on experiences with insulin injection devices [34].

Two of the items included in the new questionnaires are similar to TRIM-D Device items (confidence that the device provides the correct dose, and confidence that the device is being used correctly). However, the TRIM-D Device includes items that are not applicable to patients receiving non-insulin injectable treatment. For example, the item “adjust your medication for small dose changes” is not applicable to the GLP-1 RA injection devices that do not require dose adjustments. Similarly, the TRIM-D Device item “keep your device functioning properly” is not applicable to single use devices such as the dulaglutide pen. Also, the weekly GLP-1 RAs would not typically need to be injected in public. Therefore, the TRIM-D Device item on “using your device in public” would likely be irrelevant for patients treated with the longer acting GLP-1 RAs. Furthermore, some important features that differentiate among GLP-1 RA injection processes (e.g., injection preparation requirements relating to mixing the medication, variations in dose frequency, flexibility with regard to dose timing, requirements for needle handling) are not included in insulin-focused PRO instruments such as the TRIM-D Device. In sum, despite some overlap, the new PRO instruments are notably different from the previously available insulin-focused questionnaires.

Qualitative studies such as this one have several limitations that should be acknowledged. Items for the two new questionnaires were developed primarily based on the relatively small sample of 32 patients, and generalizability to other samples is not known. Although an effort was made to recruit a diverse group of patients from three countries, these 32 patients should not be considered completely representative of the broader population of individuals with type 2 diabetes. No cultural or geographic differences in perceptions of injection devices were apparent in the current sample. However, it is possible that samples with different demographic or clinical characteristics (e.g., age, geographic region, race/ethnicity, diabetes treatment history, symptom severity) could have different opinions and priorities relating to injection devices. For example, only two participants in this study were Asian (one in the concept elicitation and one in the cognitive interviews), and it is not known whether perceptions of non-insulin injection devices would be different in an Asian population. Future research in larger and more diverse samples may examine the broader generalizability of the resulting questionnaires.

Another limitation of the current study is that data were not collected on detailed clinical characteristics of the sample, such as diabetic complications, experience with disease self-management, detailed treatment history, and glycemic control. Therefore, it is not possible to know the extent to which this sample represents the broad range of diabetes severity. In addition, some clinical characteristics (e.g., peripheral neuropathy and loss of sensation in the fingers) could have a direct impact on one’s ability to use and prepare an injection device. The current results cannot provide insight into the impact of these clinical variables on perceptions of injection devices.

Furthermore, new non-insulin medications with new devices and treatment administration approaches will continue to be introduced [45, 46]. This study included patients treated with the full spectrum of non-insulin injectable medications for type 2 diabetes that were available at the time of data collection, including all GLP-1 RAs and pramlintide. However, it is possible that treatment administration devices used for future medications that have important characteristics that are not captured by the new questionnaires. Still, the final three global items were designed to be sufficiently general so that they could capture patients’ overall perspective of almost any diabetes injection device. Therefore, while these instruments were developed to assess perceptions of non-insulin injection devices, they may also be useful for assessing devices used to inject insulin or combinations of insulin and non-insulin medications.

It must also be emphasized that these 20-item questionnaires are draft versions, prior to psychometric validation. A follow-up study, to be reported in a subsequent article, focuses on item reduction in a larger sample, along with reliability and validity of the streamlined instruments, which are called the Diabetes Injection Device Experience Questionnaire (DID-EQ) and the Diabetes Injection Device Preference Questionnaire (DID-PQ). The qualitative research conducted in the current study is the first step toward improving measurement of patients’ experiences with devices and treatment administration approaches associated with the newer injectable medications for type 2 diabetes.

## Abbreviations

*DID-EQ*: Diabetes Injection Device Experience Questionnaire
*DID-PQ*: Diabetes Injection Device Preference Questionnaire
*DTSQ*: Diabetes treatment satisfaction questionnaire
*GLP-1 RA*: Glucagon-like peptide-1 receptor agonist
*IDSQ*: Insulin delivery system questionnaire
*IDSRQ*: Insulin delivery system rating questionnaire
*IIP-Q*: Insulin injection preference questionnaire
*IPQ-E*: Insulin pens questionnaire – evaluation
*ITSQ*: Insulin treatment satisfaction questionnaire
*PRO*: Patient-reported outcome
*PSIT*: Patient satisfaction with insulin therapy
*T2D*: Type 2 diabetes
*TRIM-D Device*: Treatment-related impact measures for diabetes and diabetes devices
*TRIM-D*: Treatment-related impact measures for diabetes
*UK*: United Kingdom
*US*: United States
